# Future Directions for Providing Conceptual Clarity Related to Context in Implementation: Comment on "Stakeholder Perspectives of Attributes and Features of Context Relevant to Knowledge Translation in Health Settings: A Multi-Country Analysis"

**DOI:** 10.34172/ijhpm.2021.173

**Published:** 2021-12-20

**Authors:** Amelia Van Pelt, Rinad S. Beidas

**Affiliations:** ^1^Department of Psychiatry, Perelman School of Medicine, University of Pennsylvania, Philadelphia, PA, USA.; ^2^Leonard Davis Institute of Health Economics, Perelman School of Medicine, University of Pennsylvania, Philadelphia, PA, USA.; ^3^Children’s Hospital of Philadelphia, Philadelphia, PA, USA.; ^4^Department of Medical Ethics and Health Policy, Perelman School of Medicine, University of Pennsylvania, Philadelphia, PA, USA.; ^5^Department of Medicine, Perelman School of Medicine, University of Pennsylvania, Philadelphia, PA, USA.; ^6^Penn Implementation Science Center at the Leonard Davis Institute of Health Economics (PISCE @ LDI), University of Pennsylvania, Philadelphia, PA, USA.; ^7^Center for Health Incentives and Behavioral Economics, University of Pennsylvania, Philadelphia, PA, USA.; ^8^Penn Medicine Nudge Unit, University of Pennsylvania Health System, Philadelphia, PA, USA.

**Keywords:** Knowledge Translation, Implementation Science, Implementation Context, Stakeholder Perspectives, Theoretical Development

## Abstract

In implementation science, contextual inquiry guides the implementation process for successful uptake of evidence-based practices. However, the conceptualization and measurement of context varies across frameworks and stakeholders. To move the field forward, future efforts to advance the understanding of context should incorporate input from implementation stakeholders through co-creation, elicit stakeholders’ perspectives in low- and middle-income countries (LMICs) to generate a more comprehensive list of determinants, and refine inconsistencies in terminology to promote research synthesis. Greater conceptual clarity and generalizability in contextual inquiry will enable improved communication and collaboration, thus facilitating a shift in focus to development and evaluation of implementation strategies to improve healthcare and health outcomes.

 To realize the promise of scientific discovery, efforts must carefully attend to the process of implementing these discoveries.^1^ Implementation science, also known as knowledge translation, has emerged as the discipline focused on systematic approaches to overcoming the research-to-practice gap. The field has grown exponentially in recent years, as illustrated by the development of over 150 theoretical frameworks, established implementation outcomes,^2^ taxonomies of implementation strategies,^3,4^ and over 600 determinants of practice^5^ (ie, factors that might serve as barriers or facilitators in the implementation process). The core approaches within implementation science focus on (1) engaging in contextual inquiry to understand the context for implementation, (2) designing and selecting implementation strategies based upon this inquiry, and (3) testing the effectiveness of the selected implementation strategies.^6^ The first step of contextual inquiry involves employing mixed-methods approaches to understand the context in which implementation will occur and the fit between the innovation and that context. This contextual inquiry is essential because context guides the implementation process for successful uptake of evidence-based practices. However, despite the recognition of the importance of understanding context, the conceptualization and measurement of “context” varies across studies and existing frameworks, which presents a challenge for the advancement of implementation science. To advance conceptual knowledge in implementation science, theoretical development needs to focus on the iterative process of theorizing that builds upon developed theory.^7,8^ The definition of context and related contextual determinants are not static. The study by Squires et al provides an example of engaging in iterative processes for development of contextual frameworks.^9^

 To address the lack of consistency in definition of concept and minimal stakeholder input in conceptualization, Squires et al elicited health system stakeholders’ perspectives to further elucidate the idea of context in implementation research. Through semi-structured interviews with implementation stakeholders (eg, implementation practitioners) and implementation science researchers in Canada, Australia, the United States, and the United Kingdom, they compiled a comprehensive list of 16 contextual attributes (ie, domains) comprised of 66 unique features (ie, specific constructs), including: patient characteristics (eg, patient demographics), health professional characteristics (eg, experience), collaboration (eg, partnerships), culture (eg, organizational culture), evaluation (eg, routinized feedback), facility characteristics (eg, geography), financial considerations (eg, funding model), governance (eg, departmental approval), leadership (eg, champions), organizational readiness for change (eg, buy-in), professional role (eg, job autonomy), resource access (eg, equipment), system features (eg, organizational changes), work structure (eg, workflow), political climate (eg, political climate generally), and regulatory and legislative standards (eg, legal). Participants from all countries described the importance of at least one feature for each of the attributes, indicating consistency across settings. These findings offer conceptual clarity regarding implementation context from the stakeholder lens while highlighting opportunities to further advance the field, as outlined below.

## Implications for Advancing Implementation Science and Future Directions

 First, the research amplifies the need to include stakeholders’ perspectives involved in implementation in the development of implementation science frameworks. A recent review on the development of implementation determinants frameworks revealed that current contextual frameworks were conceptualized through literature review of empirical studies, authors’ own implementation experiences, or review of existing theory, notably lacking input from stakeholders.^10^ In the study by Squires et al, the authors describe that only implementation stakeholders discussed the feature of “provincial responsibility” related to regulatory and legislative standards. Researchers did not posit this determinant. Thus, to yield a more robust understanding of contextual factors, and implementation science theory in general, co-creation^11^ through engagement of a range of stakeholders in different roles and functions for the development of a comprehensive repository of attributes is paramount.

 Second, the study by Squires et al underscores the need to include stakeholders’ perspectives in low- and middle-income countries (LMICs) for framework development. Implementation science frameworks aim to provide common terminology and conceptual clarity across settings, but important features and attributes vary across settings. For example, researchers have suggested adaptations to the Consolidated Framework for Implementation Research (CFIR) to include additional constructs better-suited to resource-limited settings and LMICs.^12^ The current study only included participants in four high-income countries, which limits the generalizability of the findings. To advance efforts to provide conceptual clarity, future research should elicit perspectives on context from stakeholders in LMICs. Although some determinants may only apply to LMICs, this elicitation of input would contribute to a more comprehensive list of contextual factors.

 Third, the findings emphasize the need for consistent terminology in implementation science. When comparing the results to the comprehensive Tailored Implementation for Chronic Diseases’ checklist,^5^ a comprehensive checklist for the identification of implementation determinants, Squires et al propose a new contextual attribute for consideration: facility characteristics. Facility characteristics refer to the geography, type, and size of the facility. However, similar concepts exist in other leading implementation science frameworks. For example, the CFIR^13^ includes a domain for the inner setting, referring to characteristics of the implementing organization. Although CFIR does not explicitly articulate facility characteristics as a construct, researchers often conceptualize characteristics of a facility under this domain. The current work offers an important insight by emphasizing the importance of this attribute from the stakeholders’ perspective but illustrates an opportunity for improvement in clarity. Inconsistent terminology in implementation science can hinder the advancement of knowledge by limiting the synthesis and application of concepts. Thus, a consolidation of existing implementation determinant frameworks is needed to refine terminology.^14^ Although consolidation may lose the nuance in the phrasing of a contextual determinant, consolidation will advance the field of implementation science by contributing to fewer, more comprehensive lists of contextual factors and effectively promoting collaboration and communication among researchers and stakeholders.

## Conclusion

 Overall, the work provides conceptual clarity around the idea of “context” with regard to knowledge translation and implementation science. The findings contribute to a common list of contextual factors and offer an advancement on previous work. However, to create generalizability in the understanding of context, and implementation science in general, future efforts should include the input of stakeholders across settings and refine inconsistencies in terminology. In turn, greater generalizability on the concept of context will enable better communication and collaboration between implementation researchers and stakeholders, thus facilitating a shift in focus to development and evaluation of implementation strategies to improve health outcomes.

## Ethical issues

 Not applicable.

## Competing interests

 RSB receives royalties from Oxford University Press. RSB has provided consultation to United Behavioral Health. RSB serves on the Clinical and Scientific Advisory Board for Optum Behavioral Health.

## Authors’ contributions

 AVP and RSB contributed to the concept. AVP drafted the manuscript, and RSB provided critical revision of content. Both authors have read and approved the final manuscript.

## Funding

 This work was supported by the National Institutes of Health (P50 CA244690), the Penn Implementation Science Center at the Leonard Davis Institute of Health Economics (PISCE@LDI), and the Penn CFAR ISPHERE Scientific Working Group, an NIH-funded (P30 045088) program.

## References

[1] Morris ZS, Wooding S, Grant J (2011). The answer is 17 years, what is the question: understanding time lags in translational research. J R Soc Med.

[2] Proctor E, Silmere H, Raghavan R (2011). Outcomes for implementation research: conceptual distinctions, measurement challenges, and research agenda. Adm Policy Ment Health.

[3] Powell BJ, Waltz TJ, Chinman MJ (2015). A refined compilation of implementation strategies: results from the Expert Recommendations for Implementing Change (ERIC) project. Implement Sci.

[4] Michie S, Richardson M, Johnston M (2013). The behavior change technique taxonomy (v1) of 93 hierarchically clustered techniques: building an international consensus for the reporting of behavior change interventions. Ann Behav Med.

[5] Flottorp SA, Oxman AD, Krause J (2013). A checklist for identifying determinants of practice: a systematic review and synthesis of frameworks and taxonomies of factors that prevent or enable improvements in healthcare professional practice. Implement Sci.

[6] Lane-Fall MB, Curran GM, Beidas RS (2019). Scoping implementation science for the beginner: locating yourself on the “subway line” of translational research. BMC Med Res Methodol.

[7] Kislov R, Pope C, Martin GP, Wilson PM (2019). Harnessing the power of theorising in implementation science. Implement Sci.

[8] Sales AE, Barnaby DP, Rentes VC. Letter to the editor on “the implementation research logic model: a method for planning, executing, reporting, and synthesizing implementation projects” (Smith JD, Li DH, Rafferty MR. the implementation research logic model: a method for planning, executing, reporting, and synthesizing implementation projects. Implement Sci 2020;15 (1):84. 10.1186/s13012-020-01041-8. PMC7523057 32988389

[9] Squires JE, Hutchinson AM, Coughlin M (2022). Stakeholder perspectives of attributes and features of context relevant to knowledge translation in health settings: a multi-country analysis. Int J Health Policy Manag.

[10] Nilsen P, Bernhardsson S (2019). Context matters in implementation science: a scoping review of determinant frameworks that describe contextual determinants for implementation outcomes. BMC Health Serv Res.

[11] Metz A. Implementation Brief: The Potential of Co-Creation in Implementation Science. https://nirn.fpg.unc.edu/sites/nirn.fpg.unc.edu/files/resources/NIRN-Metz-ImplementationBreif-CoCreation.pdf. Published 2015.

[12] Means AR, Kemp CG, Gwayi-Chore MC (2020). Evaluating and optimizing the consolidated framework for implementation research (CFIR) for use in low- and middle-income countries: a systematic review. Implement Sci.

[13] Damschroder LJ, Aron DC, Keith RE, Kirsh SR, Alexander JA, Lowery JC (2009). Fostering implementation of health services research findings into practice: a consolidated framework for advancing implementation science. Implement Sci.

[14] Davis M, Beidas RS (2021). Refining contextual inquiry to maximize generalizability and accelerate the implementation process. Implement Res Pract.

